# Epidermal Growth Factor Removal or Tyrphostin AG1478 Treatment Reduces Goblet Cells & Mucus Secretion of Epithelial Cells from Asthmatic Children Using the Air-Liquid Interface Model

**DOI:** 10.1371/journal.pone.0129546

**Published:** 2015-06-09

**Authors:** Jeremy C. Parker, Isobel Douglas, Jennifer Bell, David Comer, Keith Bailie, Grzegorz Skibinski, Liam G. Heaney, Michael D. Shields

**Affiliations:** 1 Centre for Infection and Immunity, Health Sciences Building, Queen’s University Belfast, Belfast, Northern Ireland; 2 Royal Belfast Hospital for Sick Children, Belfast, Northern Ireland; University of Alabama at Birmingham, UNITED STATES

## Abstract

**Rationale:**

Epithelial remodelling in asthma is characterised by goblet cell hyperplasia and mucus hypersecretion for which no therapies exist. Differentiated bronchial air-liquid interface cultures from asthmatic children display high goblet cell numbers. Epidermal growth factor and its receptor have been implicated in goblet cell hyperplasia.

**Objectives:**

We hypothesised that EGF removal or tyrphostin AG1478 treatment of differentiating air-liquid interface cultures from asthmatic children would result in a reduction of epithelial goblet cells and mucus secretion.

**Methods:**

In Aim 1 primary bronchial epithelial cells from non-asthmatic (n = 5) and asthmatic (n = 5) children were differentiated under EGF-positive (10ng/ml EGF) and EGF-negative culture conditions for 28 days. In Aim 2, cultures from a further group of asthmatic children (n = 5) were grown under tyrphostin AG1478, a tyrosine kinase inhibitor, conditions. All cultures were analysed for epithelial resistance, markers of differentiation using immunocytochemistry, ELISA for MUC5AC mucin secretion and qPCR for MUC5AC mRNA.

**Results:**

In cultures from asthmatic children the goblet cell number was reduced in the EGF negative group (p = 0.01). Tyrphostin AG1478 treatment of cultures from asthmatic children had significant reductions in goblet cells at 0.2μg/ml (p = 0.03) and 2μg/ml (p = 0.003) as well as mucus secretion at 2μg/ml (p = 0.04).

**Conclusions:**

We have shown in this preliminary study that through EGF removal and tyrphostin AG1478 treatment the goblet cell number and mucus hypersecretion in differentiating air-liquid interface cultures from asthmatic children is significantly reduced. This further highlights the epidermal growth factor receptor as a potential therapeutic target to inhibit goblet cell hyperplasia and mucus hypersecretion in asthma.

## Introduction

Asthma is a disease that normally begins during childhood [1, 2] progressing into adulthood with airways remodelling including goblet cell hyperplasia due to chronic inflammation. Differentiated air-liquid interface (ALI) cultures of primary bronchial epithelial cells from asthmatic children express a goblet cell hyperplasia [3]. MUC5AC is the major mucus forming mucin produced mainly by goblet cells [4–6]. We have previously shown that the Th2 cytokine interleukin 13 (IL-13) caused goblet cell hyperplasia, decreased ciliated cell number and increased mucus secretion in differentiating cultures of healthy epithelial cells while IL-13 stimulation of differentiating ALI cultures from asthmatic children also showed increased goblet cell number [7]. However under unstimulated conditions where an inherent goblet cell hyperplasia existed we detected no IL-13 mRNA or protein. We deduced that while IL-13 can induce an asthmatic phenotype, it was unlikely to be the cause of the inherent goblet cell hyperplasia seen in unstimulated ALI cultures from asthmatic children [7]. This lead us to consider epidermal growth factor (EGF) and its receptor (EGFR) which have been shown to induce MUC5AC via ERK pathway activation as ERK inhibition effectively reduces MUC5AC mRNA production and promoter activity [8]. Current asthma therapies focus on reducing inflammation and relieving airway hyperresponsiveness (AHR) but these do not address airways remodelling. There have been attempts to target mucus hypersecretion using mucoactive drugs however these have provided minimal benefit [9].

EGFR is highly expressed throughout many tissues and functions in a variety of processes including proliferation, differentiation and cell survival [10]. In asthma, overexpression of EGF/EGFR correlates with disease severity and epithelial injury [11, 12]. In sputum from asthmatic patients during and following an acute asthma attack, EGF levels were highly elevated and prolonged [13]. EGF has been shown to induce altered epithelial basal differentiation towards an EMT-like phenotype commonly seen in smokers [14] and in other studies EGFR has been implicated in goblet cell hyperplasia [15, 16].

In murine models of asthma, Egf/Egfr was shown to be important in goblet cell differentiation [17, 18] while under ovalbumin challenge in Brown Norway rats, increased airway epithelial cell proliferation and goblet cell differentiation was observed via Egfr activation [19]. Egfr inhibition reduced AHR and inflammation in ovalbumin challenged animals [19]. By specifically targeting Egfr with tyrphostin AG1478 (AG1478), a tyrosine kinase inhibitor, prior to ovalbumin challenge, goblet cell numbers were reduced to basal levels identifying Egfr as a therapeutic target [19].

These studies used models which are challenged with an exogenous agent to induce the asthmatic phenotype during which inhibition of EGFR is then assessed. To our knowledge this preliminary study is the first to look at the effects of an EGFR tyrosine kinase inhibitor on human pediatric asthmatic epithelium which demonstrates a goblet cell hyperplasia *ex vivo* [3]. EGF/EGFR has a role to play in the disequilibrium of the bronchial epithelium in asthma and is considered a promising target for pharmacotherapy [4]. The aim of this study is to assess whether removal of EGF or treatment with AG1478 will result in a reduction of goblet cell number and mucus secretion in epithelium from asthmatic children.

### Hypothesis

We hypothesised that chronic removal of EGF or treatment with AG1478 would reduce the inherent goblet cell hyperplasia and mucus secretion in differentiating ALI cultures from asthmatic children.

### Aims

Our first aim (Aim 1) was to sample and differentiate bronchial epithelial cells from 5 non-asthmatic and 5 asthmatic children in the presence or absence of EGF to assess if the removal of EGF resulted in a reduction in the numbers of goblet cells and mucus secretion.

Our second aim (Aim 2) was to sample a further 5 asthmatic children and differentiate their bronchial epithelial cells in the absence of EGF or in the presence of EGF +/- AG1478 to assess goblet cell number and mucus secretion.

## Methods

### Subjects

Children 13 years or less attending elective surgical procedures at the Royal Belfast Hospital for Sick Children were recruited. A clinician-administered semi-structured pro-forma [20] was used to record the clinical history. Recruited children were either doctor diagnosed asthma or non-asthmatic controls who had never wheezed (Table 1). Written informed parental consent was obtained. This study was approved by the Office of the Research Ethics Committees of Northern Ireland.

**Table 1:** Clinical Details of Subjects.

Clinical Status	Gender (M/F)	Age (y)	Serum IgE Concentration (kU/L)	Medication	Cohort
Non-asthmatic	M	3	32	None	1
Non-asthmatic	M	12	14	None	1
Non-asthmatic	M	12	25	None	1
Non-asthmatic	F	11	18	None	1
Non-asthmatic	M	1	3	None	1
Asthmatic	M	11	1071	Beta 2 agonist as required, alone	1
Asthmatic	M	12	780	Adrenaline auto-injector, ICS+LAB2A, LTA	1
Asthmatic	M	7	27	ICS+LAB2A, LTA	1
Asthmatic	M	12	910	ICS+LAB2A, LTA	1
Asthmatic	F	4	209	ICS+LAB2A, LTA, antihistamine.	1
Asthmatic	F	1	2	ICS, LTA	2
Asthmatic	M	6	1908	ICS+LAB2A	2
Asthmatic	F	1	2	ICS	2
Asthmatic	F	13	313	ICS	2
Asthmatic	F	13	304	ICS+LAB2A,antihistamine	2

NB/. ICS–Inhaled corticosteroid; LAB2A –Long-acting beta 2 agonist; LTA–Leukotriene antagonist.

### Isolation of primary bronchial epithelial cells for Differentiated ALI cultures

Non-bronchoscopic brushings were used to sample bronchial epithelial cells as previously described [3,7,21] and samples were tested for the presence of viral contamination [22]. Primary bronchial epithelial cells were expanded and seeded at 0.8x10^5^ cells per membrane and grown as previously described [3,7,23,24]. All cells from subjects used in this study were differentiated at passage 2.

### Stimulation of Differentiating ALI cultures

For Aim 1, differentiating ALI cultures from non-asthmatic (n = 5) and asthmatic (n = 5) children were fed every other day for 28 days with EGF+ve (10ng/ml EGF) or EGF-ve (EGF supplement omitted from the media) culture media.

For Aim 2, differentiating ALI cultures from a further group of asthmatic (n = 5) children were fed with either EGF+ve, EGF-ve or EGF+ve medium supplemented with AG1478 (0.2 or 2μg/ml) (Cayman Chemical Co., MI, USA).

### Measurement of Transepithelial Electrical Resistance (TEER)

TEER was measured weekly using an EVOM meter and an ENDOHM-12 chamber (World Precision Instruments, FL, USA) [25].

### Quantification of goblet and ciliated cells using immunocytochemistry

Cultures were trypsinised with cytospin slides being made as previously described [3, 7]. Goblet cells and ciliated cells were identified using a mouse monoclonal antibody for MUC5AC (clone 45M1) (1:200 dilution) and a mouse monoclonal antibody for acetylated alpha tubulin (6–11 B-1) (1:700 dilution) respectively (Abcam, UK). Slides were anonymized and counts are represented as a mean % of 500 cells counted per slide.

### ELISA measuring MUC5AC mucin secretion

Apical secretion of MUC5AC mucin from ALI cultures was measured using an in-house MUC5AC ELISA [3, 7] adapted from Takeyama and colleagues [17].

### RNA extraction, cDNA synthesis and qPCR for MUC5AC

Total RNA was extracted using an RNeasy Mini kit (Qiagen, Crawley, UK) followed by cDNA synthesis performed using GoScript Reverse Transcription System (Medical Supply Co., Republic of Ireland). DNA amplification was carried out using the Fast Start Universal SYBR Green Master (Rox) (Roche, UK) according to all manufacturers’ instructions. Samples were assayed in duplicate on an Agilent Mx3005P 96-well detection plate system.

### Statistical Analysis

Descriptive data was expressed as mean (standard deviation, SD) or median (interquartile range, IQR). We used one and two-way repeated measures ANOVA while, when appropriate, simultaneously adjusted for differing total cell counts (covariate) in each culture.

If in the overall model p≤ 0.05 we performed *a priori* planned comparisons using unadjusted paired t-tests. Analysis was carried out using JMP version 11 (SAS, Cary, North Carolina, USA) and GraphPad PRISM 5 (CA, USA). A p-value of <0.05 was taken as statistically significant.

All methods are described in detail in the supplementary information (S1 Methods).

## Results

### Patient Characteristics

Asthmatic children were similarly aged compared to those without asthma (asthmatic: mean age of 8 years (SD 4.8), range 1–13 years, non-asthmatic: mean age of 7.8 years (SD 5.4), range 1–12 years, p = 0.94). Median total serum IgE concentrations were higher in asthmatic (median = 308 (IQR 21–950), range 2–1908 kU/L) compared with non-asthmatic patients (median = 18 (IQR 3–29), range 2–32 kU/L). 50% of the asthmatic group were male whereas 80% of the non-asthmatic group were male. Asthmatic children studied in Aim 1 and Aim 2 had similar ages and similar total IgE levels.

### ALI cultures maintained epithelial resistance measurements in the absence of EGF and during AG1478 treatment throughout the differentiation process

**Fig 1:**
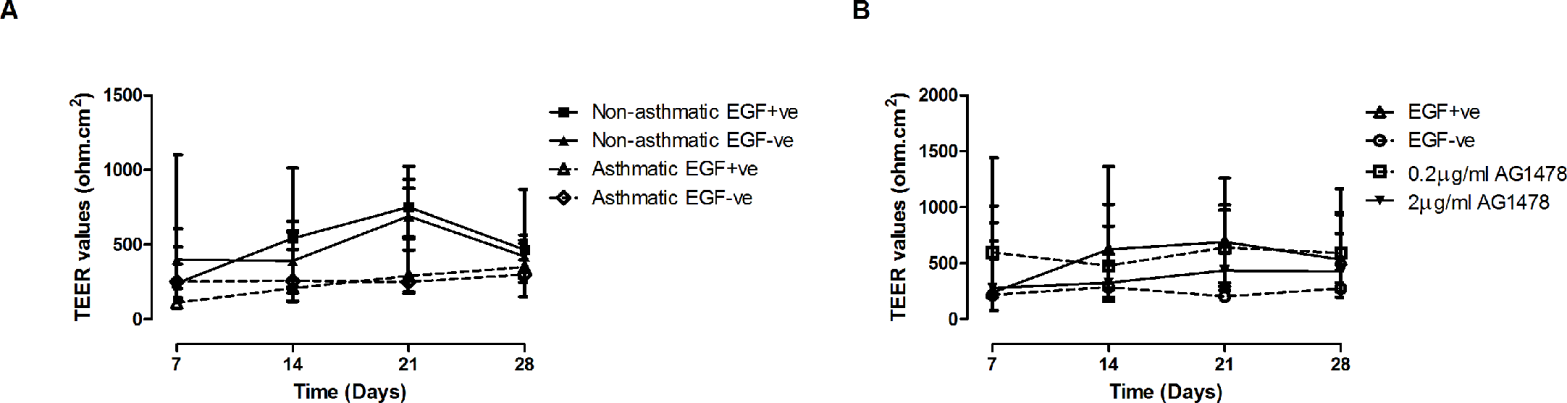
Measurement of Epithelial Resistance. Transepithelial Electrical Resistance (TEER) measurements on days 7, 14, 21 & 28 of ALI culture. (A) TEER measurements over 28 days at ALI for asthmatic and non-asthmatic cultures in the presence or absence of EGF (10ng/ml) (n = 5) and (B) in the presence or absence of EGF +/- AG1478 (0.2 or 2μg/ml) (n = 5). Values are expressed as median with interquartile range. Data was analysed using a Kruskal-Wallis test with Dunn’s multiple comparison post hoc test.

ALI cultures from non-asthmatic and asthmatic children in Aim 1 of the study displayed no significant difference in TEER values during differentiation in the absence or presence of EGF, as was also the case in Aim 2 of the study in cultures from asthmatic children stimulated with AG1478 (Fig 1A and 1B).

### ALI cultures proliferate at different rates in the absence of EGF

**Fig 2:**
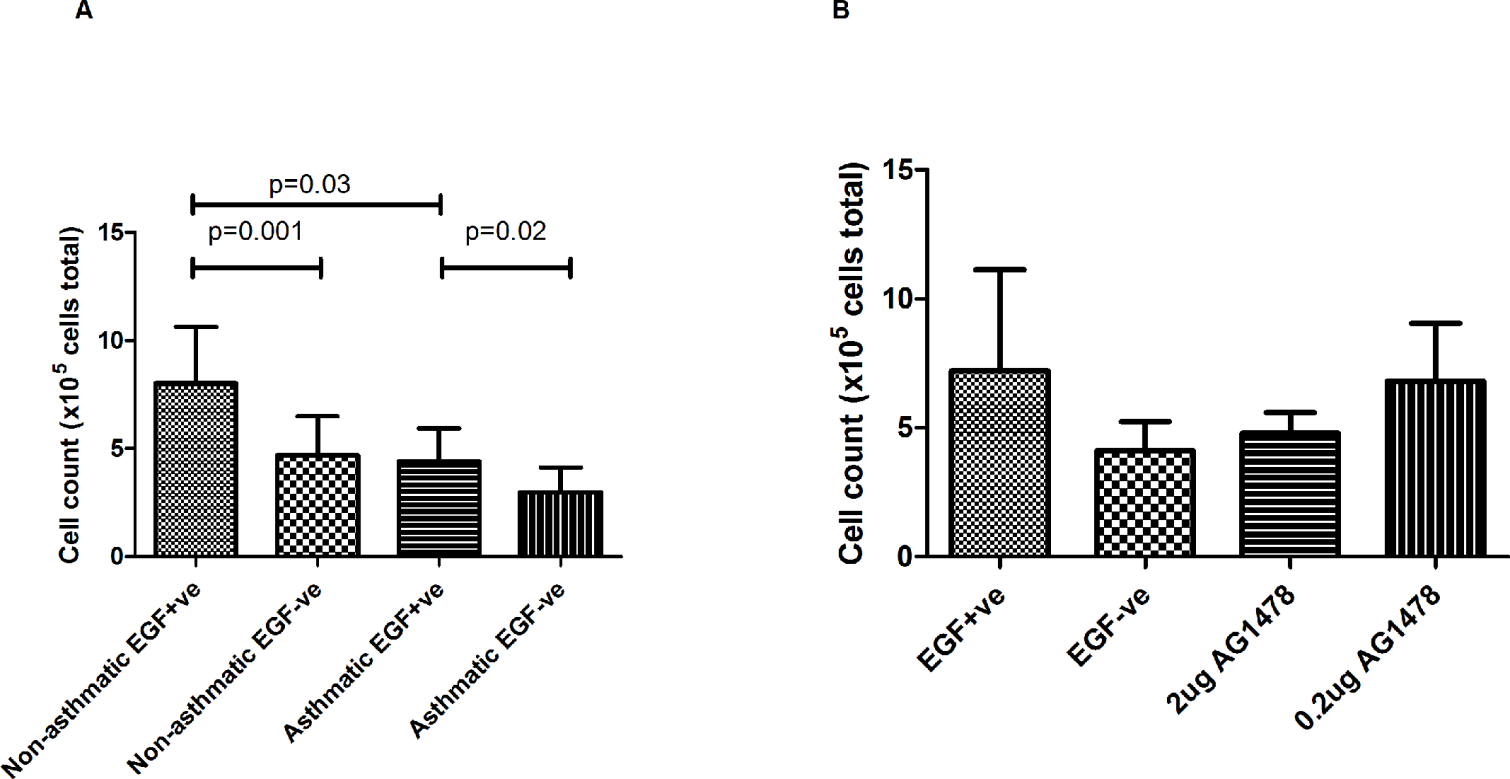
Total Cell Count. Total cell count (x10^5^) on day 28 of ALI culture. (A) Asthmatic and non-asthmatic cultures in the presence or absence of EGF (10ng/ml) (n = 5). Data analysed using a 2-way repeated measures ANOVA with planned unadjusted t-tests. (B) Asthmatic cultures in the presence or absence of EGF +/- AG1478 (0.2 or 2μg/ml) (n = 4). Data analysed using a 1-way repeated measures ANOVA. Values are expressed as mean (SD).

With respect to total cell number on day 28 of Aim 1 EGF+ve cultures from non-asthmatic children (mean 8.0x10^5^ (SD 2.6) cells/well) differed significantly compared with the EGF-ve group (mean 4.7x10^5^ (SD 1.8) cells/well, p = 0.001) and the asthmatic EGF+ve group (mean 4.4x10^5^ (SD 1.5) cells/well, p = 0.03,) in total cell number (Fig 2A). The asthmatic EGF+ve group (mean 4.4x10^5^ (SD 1.5) cells/well) had significantly higher cell numbers at day 28 compared with the asthmatic EGF-ve group (mean 3.0x10^5^ (SD 1.1) cells/well, p = 0.02) (Fig 2A). In Aim 2 of the study, ALI cultures from asthmatic children stimulated with AG1478 exhibited no significant differences between EGF+ve controls and EGF-ve or AG1478 stimulations (Fig 2B).

### Removal of EGF or AG1478 treatment during differentiation reduces the percentage of goblet cells and mucus secretion with no effect on MUC5AC mRNA in ALI cultures

**Fig 3:**
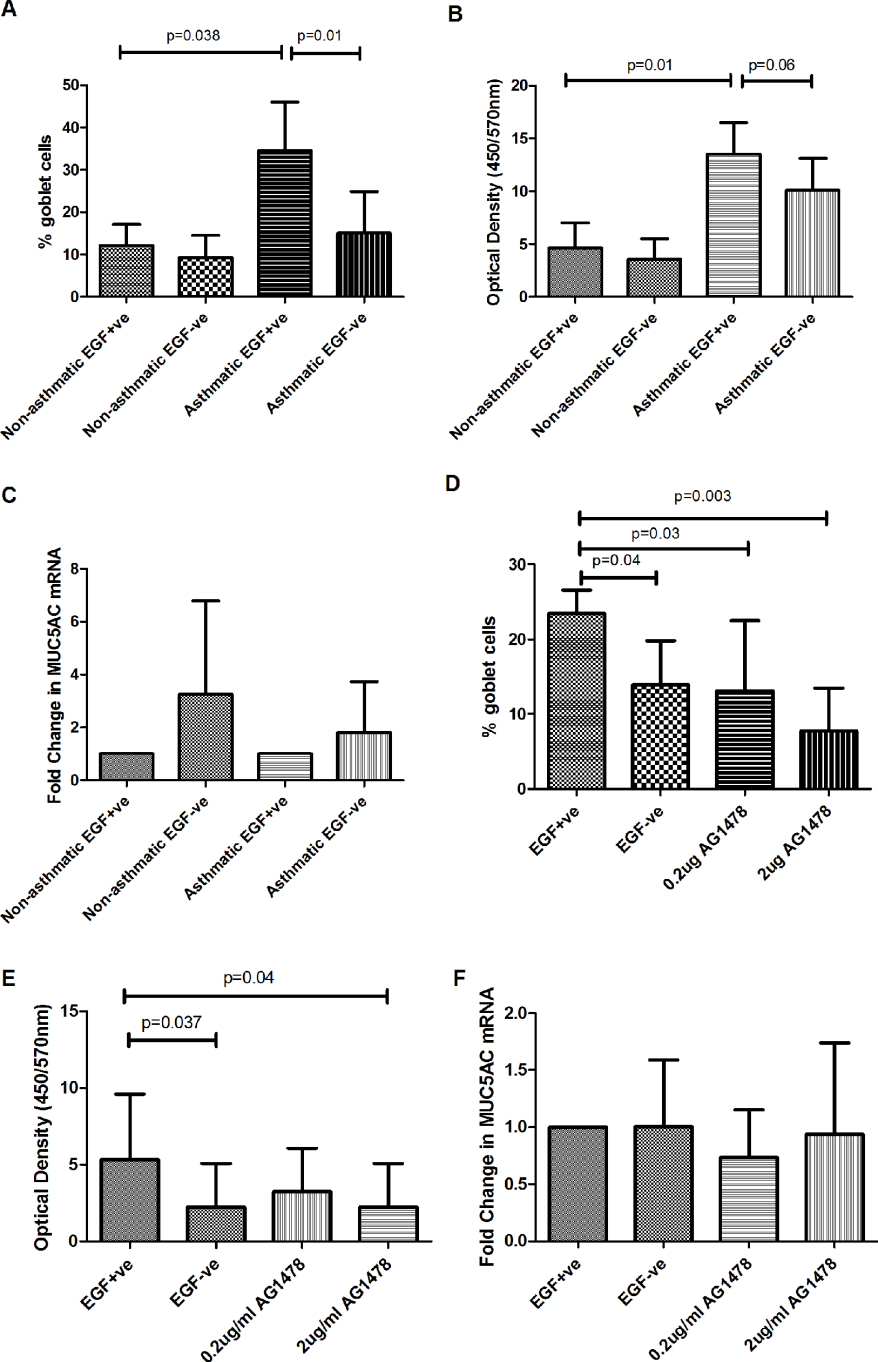
Goblet Cell Quantification, MUC5AC Secretion Measurements and MUC5AC qPCR. (A) Goblet cells expressed as a percentage of total in asthmatic and non-asthmatic cultures in the presence or absence of EGF (10ng/ml) (n = 5). Data analysed using a 2-way repeated measures ANOVA with planned t-tests adjusted for total cell number. (B) Mucus secretion presented as average relative optical density of apical washings measured for MUC5AC mucin on day 28 of ALI culture in the presence or absence of EGF (10ng/ml) (n = 5). Data analysed using a two-way repeated measures mixed (random and fixed) effects design. (C) Fold change of MUC5AC mRNA in asthmatic and non-asthmatic ALI cultures in the presence or absence of EGF (10ng/ml) (n = 5). Data was analysed using a 2-way repeated measures ANOVA. (D) Goblet cells expressed as a percentage of total in asthmatic cultures in the presence or absence of EGF +/- AG1478 (0.2 or 2μg/ml) (n = 5). Data was analysed using a 1-way repeated measures ANOVA with planned unadjusted t-tests. (E) Mucus secretion presented as average relative optical density of apical washings measured for MUC5AC mucin on day 28 of ALI culture in the presence or absence of EGF +/- AG1478 (0.2 or 2μg/ml) (n = 5). Data was log transformed due to skewing and was analysed using a 1-way repeated measures ANOVA with a Tukey-Kramer multi-comparison test. (F) Fold change of MUC5AC mRNA in asthmatic ALI cultures in the presence or absence of EGF +/- AG1478 (0.2 or 2 μg/ml) (n = 5). Data was analysed using a 1-way repeated measures ANOVA. Values are expressed as mean (SD).

At the end of Aim 1, tests for simple effects (no interaction effect included) showed that in the presence of EGF the mean percentage goblet cells for asthmatic cultures was statistically significantly higher than for non-asthmatics (mean %GC asthmatic 34.5% (SD 11.4) versus mean %GC non-asthmatic 12.0% (SD 5.0), p = 0.038). Similarly in the presence of EGF the mean percentage goblet cells for asthmatic cultures was statistically significantly higher compared with EGF-ve cultures (mean %GC asthmatic 34.5% (SD 11.4) versus mean %GC 15.0% (SD 9.8), p = 0.01) (Fig 3A). Importantly, the clinical grouping (asthma versus non-asthmatic) by EGF conditions (EGF+ve versus EGF-ve) interaction on goblet cell percentage was statistically significant (p = 0.005) indicating that asthmatic samples behaved differently compared with non-asthmatics with respect to the presence or absence of EGF during differentiation.

The clinical grouping (asthmatic versus non-asthmatics) and EGF conditions (as detailed above) interaction for MUC5AC mucin secretion on Day 28 was not statistically significant (p = 0.4) and therefore was not included. Tests for simple effects (no interaction included) showed that the mean MUC5AC absorbance for cultures from asthmatic children was significantly higher than for non-asthmatic children (p = 0.01) and was higher (but not statistically significant) in EGF+ve versus EGF-ve cultures (p = 0.06) (Fig 3B). Post-hoc contrast showed that it was cultures from asthmatic children (EGF+ve and EGF-ve) which secreted higher levels of MUC5AC mucin compared with non-asthmatic children (EGF+ve and EGF-ve) (Fig 3B). ALI cultures showed no significant difference in MUC5AC mRNA between the non-asthmatic EGF-ve group (3.3 fold increase) and the non-asthmatic EGF+ve group (mean 1.0 fold increase) as was also the case with the asthmatic EGF-ve group and the asthmatic EGF+ve group (Fig 3C).

ALI cultures from asthmatic children in Aim 2 of the study displayed a lower percentage of goblet cells in the unstimulated EGF+ve group (mean 23.4% SD 3.1) reflecting patient variability (Fig 3D). Within the dataset for Fig 3D there was an obvious visible outlier which we have removed in order than any true effect was not masked or skewed by the outlying data point. Cultures from the EGF-ve group displayed a significant reduction in the percentage of goblet cells compared with the EGF+ve group (mean 13.9% SD 5.9, p = 0.04). ALI cultures from the 0.2μg/ml (mean %GC 13.1% (SD 9.4), p = 0.03) and 2μg/ml (mean %GC 7.7% (SD 5.7), p = 0.003) AG1478 group also exhibited a significant reduction in goblet cells when compared with the EGF+ve group (mean 23.4% SD 3.1) (Fig 3D).

In AG1478 treatment of ALI cultures from asthmatic children we observed a significant reduction in the amount of secreted MUC5AC mucin in the EGF-ve group (p = 0.037) and the 2μg/ml AG1478 group (p = 0.04) compared with the EGF+ve group (Fig 3E). In ALI cultures from asthmatic children differentiated under stimulation with AG1478 we saw no significant difference in MUC5AC mRNA between EGF+ve and EGF-ve or AG1478 stimulations (Fig 3F).

### Removal of EGF or AG1478 treatment does not have a detrimental effect on ciliated cells

**Fig 4:**
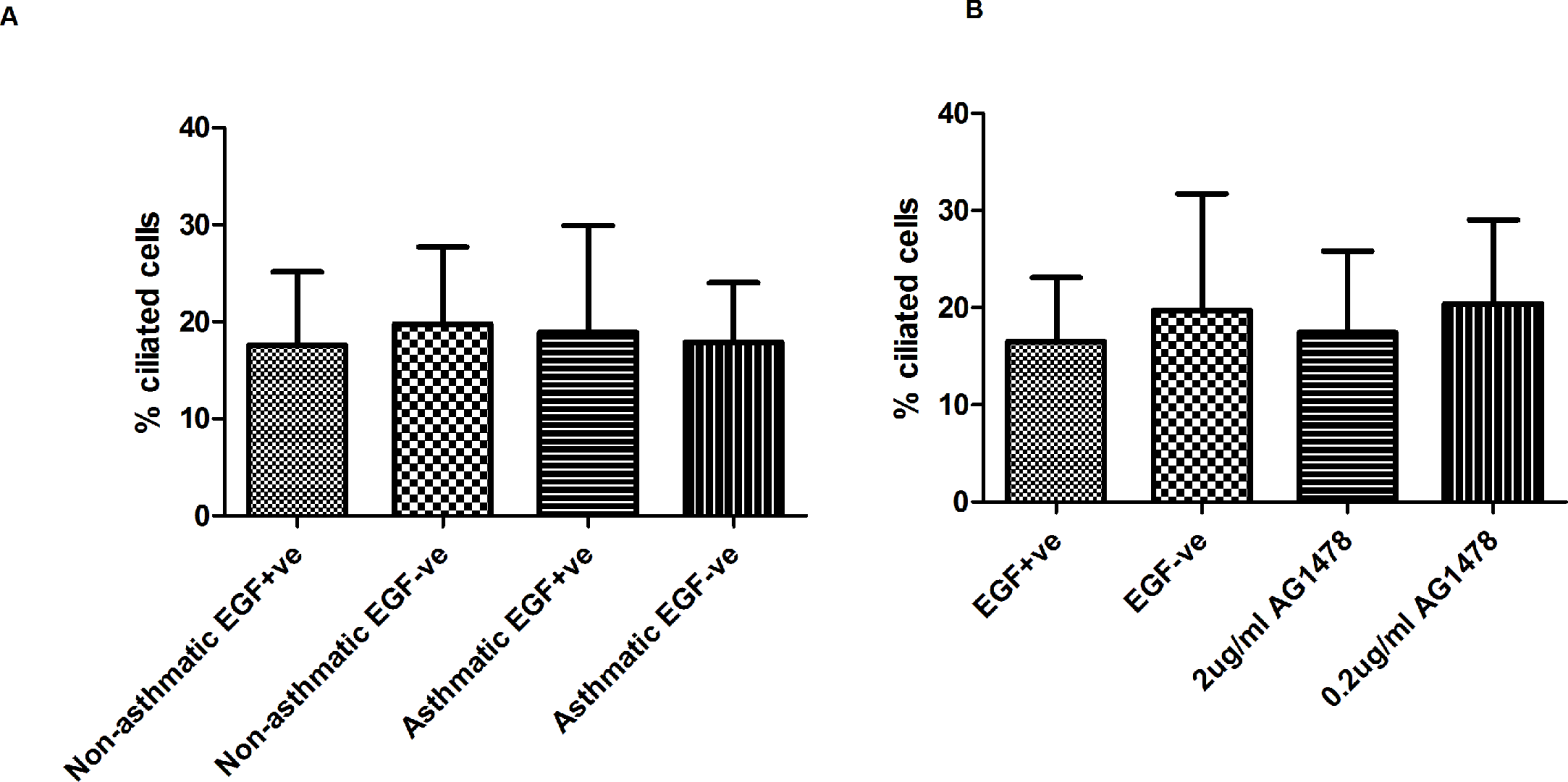
Ciliated Cell Quantification. Quantification of ciliated cells on day 28 of ALI culture expressed as a percentage of total on day 28. (A) Ciliated cells expressed as a percentage of total in the presence or absence of EGF (10ng/ml) (n = 5). Data was analysed using a 2-way repeated measures ANCOVA. (B) in the presence or absence of EGF +/- AG1478 (0.2 or 2μg/ml) (n = 5). Data was analysed using a 1-way repeated measures ANOVA. Values are expressed as mean (SD).

In Aim 1 of the study we found no statistical difference between the mean percentage ciliated cells and either clinical grouping (p = 0.8), EGF conditions (p = 0.68) and their interaction (p = 0.55) (Fig 4A). ALI cultures from asthmatic children in Aim 2 of the study displayed similar numbers of ciliated cells during differentiation with AG1478 (Fig 4B).

## Discussion

In Aim 1 of this preliminary study we confirmed that removal of EGF during differentiation of ALI cultures from asthmatic children significantly reduced the goblet cell number (p = 0.01) and reduced MUC5AC mucin secretion albeit not significantly (p = 0.06) (Fig 3A and 3B). Other morphological assessments demonstrated that epithelial resistance, MUC5AC mRNA and ciliated cell number remained unchanged in asthmatic and non-asthmatic groups (Figs 1, 3C, 3F and 4). This suggests that EGF removal resulted in a specific change related to goblet cell differentiation rather than as a result of an overall morphological change of the epithelium.

We then sampled a further five asthmatic children for Aim 2 of the study which involved using tyrphostin AG1478, a tyrosine kinase inhibitor. We noted that in this group of asthmatic patients there were a lower mean percentage of goblet cells compared with the group of cultures from asthmatic children in Aim 1 (mean %GC 23.4% (SD 3.1) versus mean % GC 34.5% (SD 11.4), respectively). This difference is also reflected in the MUC5AC ELISA data between Aims 1 and 2. However in Aim 2 there was a statistical difference in the percentage of goblet cells (p = 0.04) and the MUC5AC secretion (p = 0.037) between the EGF+ve and EGF-ve cultures. Although variability between cohorts exists, the changes observed follow a consistent trend. We observed a significant reduction in the goblet cell number associated with asthmatic epithelium through AG1478 treatment at 0.2μg/ml AG1478 (p = 0.04) and 2μg/ml AG1478 (p = 0.003) with a significant reduction in the amount of mucus secreted (p = 0.04) in the 2μg/ml AG1478 group (Fig 3D and 3E). The results from Aim 1 & 2 suggest the involvement of EGF/EGFR in the goblet cell hyperplasia and subsequent mucus hypersecretion that are characteristic of our asthmatic epithelial ALI cell model [3, 7].

Current asthma therapies do not address goblet cell hyperplasia or mucus hypersecretion. EGFR inhibitors such as gefitinib and erlotinib are used in lung cancer therapy where EGFR overexpression is implicated [26, 27]. Erlotinib has been used in a murine house dust mite model of asthma where it reduced goblet cell hyperplasia, airway hyperresponsiveness and inflammation [28] and gefitinib has been used in an ovalbumin challenge murine model of asthma where it reduced inflammatory cell counts and cytokines [26]. We have shown in this study using the *ex vivo* ALI model of epithelium from asthmatic children that AG1478 also reduced goblet cell hyperplasia and mucus hypersecretion. This adds further strength to the possibility that targeting EGFR may be a potential therapeutic for airway remodelling, a current unmet need in asthma treatment. However, EGFR inhibition may have undesirable side-effects including impaired response to viral, allergen and airway pollutant insults [29] but in an Egfr^-/-^ murine model no overt gross changes in lung structure following EGFR inhibition were observed [30].

Our study, though preliminary, has shown that there is potential in altering airway remodelling in asthmatic ALI cultures through EGF removal or AG1478 treatment. We suggest that further *in vitro* studies with a larger cohort of asthmatic children using the ALI model of bronchial epithelium would be the next step in determining whether EGF inhibitors, such as AG1478, could be considered as a viable therapeutic for reducing goblet cell hyperplasia and mucus hypersecretion in asthma.

Our study is limited by small sample numbers in each group due to its preliminary nature. A recent article has highlighted that obtaining lower airway samples from children is often more difficult than from adult patients which will result in small study numbers [31]. When samples are from optimal groups however the data obtained is still meaningful [31]. We believe that the results we have observed, though preliminary, are indeed real and of significant interest warranting further study of EGFR inhibitors in ALI cultures from asthmatic children.

In conclusion, to our knowledge, we are the first to show the effect of AG1478 treatment in human pediatric asthmatic epithelium which significantly reduced the goblet cell hyperplasia and mucus secretion. These results encourage further studies to consider EGF/EGFR as a potential therapeutic target capable of reducing the goblet cell hyperplasia and mucus hypersecretion inherent to asthmatic bronchial epithelium.

## Supporting Information

S1 Methods Detailed description of the methods section. (DOCX)
