# Supplementary material for: Epidermal Growth Factor Removal or Tyrphostin AG1478 Treatment Reduces Goblet Cells & Mucus Secretion of Epithelial Cells from Asthmatic Children Using the Air-Liquid Interface Model
Source: PLoS One. 2015 Jun 9;10(6):e0129546. doi: 10.1371/journal.pone.0129546 (PMC4461195; doi:10.1371/journal.pone.0129546)
Supplement: S1 Methods — (DOCX) [file pone.0129546.s001.docx]

**Epidermal Growth Factor Removal or Tyrphostin AG1478 treatment reduces goblet cells & mucus secretion of epithelial cells from asthmatic children using the air-liquid interface model**

Jeremy C Parker^1^, Isobel Douglas^2^, Jennifer Bell^1^, David Comer^1^, Keith Bailie^2^, Grzegorz Skibinski^1^, Liam G Heaney^1*^, Michael D Shields^1&2*^

**S1 Methods – Detailed description of the methods section**

**Subjects**

Children 13 years or less attending elective surgical procedures at the Royal Belfast Hospital for Sick Children were recruited. A clinician-administered semi-structured pro-forma [E1] was used to record the clinical history and recruited children were either doctor diagnosed asthma or healthy controls (Table 1). Children with doctor diagnosed asthma had other atopy (either or both allergic rhinitis and eczema and / or an elevated total serum IgE). One child with asthma also had a history of food induced anaphylaxis. Written informed parental consent was obtained. This study was approved by the Office of the Research Ethics Committees of Northern Ireland.

**Isolation of primary bronchial epithelial cells for Differentiated ALI cultures**

Non-bronchoscopic bronchial brushings were used to sample and culture bronchial epithelial cells as previously described [E2-E4]. All brush washings were analysed for viruses using a multi-viral PCR analysis and only uncontaminated cultures were used [E5]. We sampled 10 asthmatic children and 5 non-asthmatic children suitable for study.

Primary bronchial epithelial cells were seeded at a density of 0.8x10^5^ cells per membrane and grown as previously described [E2, E3, E6, E7]. All cells from subjects used in this study were grown at ALI at passage 2 in ALI medium consisting of a 50:50 mixture of Basal Airway Epithelial Cell Growth Medium (Promocell, Heidelberg, Germany) and DMEM (Invitrogen Ltd, Paisley, UK) supplemented with bovine pituitary extract (52 µg/ml), epidermal growth factor (10 ng/ml), insulin (5 μg/ml), hydrocortisone (0.5 μg/ml), epinephrine (0.5 μg/ml), transferrin (10 μg/ml), bovine serum albumin (1.5 µg/ml), penicillin/streptomycin (100 IU/ml/100 µg/ml) and retinoic acid (50 nM). For EGF+ve cultures the above media was used with a final EGF concentration of 10ng/ml. For the EGF-ve cultures the EGF supplement was omitted during media preparation. For the AG1478 supplemented media the tyrosine kinase inhibitor was added in two concentrations (0.2 & 2µg/ml) into the EGF+ve culture media (ie/. in the presence of 10ng/ml EGF).

**Stimulation of Differentiating ALI cultures**

For Aim 1 of the study, differentiating ALI cultures from non-asthmatic (n=5) and asthmatic (n=5) children were fed basolaterally every other day for 28 days with EGF+ve or EGF-ve culture media immediately following ALI creation. The apical side was washed weekly with PBS and aliquots of washing and basolateral media were stored for analysis.

For Aim 2 of the study, bronchial epithelial cells from a further group of asthmatic (n=5) children were fed with either EGF+ve, EGF-ve or EGF+ve medium supplemented with tyrophostin AG1478 (AG1478), an EGFR tyrosine kinase inhibitor (0.2 or 2µg/ml) (Cayman Chemical Co., MI, USA).

**Measurement of Transepithelial Electrical Resistance (TEER)**

TEER was measured on days 7, 14, 21 & 28 using an EVOM meter and an ENDOHM-12 chamber (World Precision Instruments, FL, USA) [E8].

**Total Cell Count**

Cells were trypsinised, stained with trypan blue and viable cells counted using a haemocytometer.

**Quantification of goblet and ciliated cells using immunocytochemistry**

Cultures were washed with PBS and trypsinised with cytospin slides being made as previously described [E2, E3]. Slides were anonymized prior to counting. Briefly, 5x10^4^cells were spun onto each slide, stained and the numbers of positive cells for each stain (mouse monoclonal anti-MUC5AC antibody for goblet cells; mouse monoclonal anti-acetylated alpha tubulin antibody for ciliated cells) (Abcam, UK) counted. These counts are represented as a mean % of 500 cells counted per slide.

**ELISA measuring MUC5AC mucin secretion**

Apical secretion of MUC5AC mucin from differentiated ALI cultures was measured using an in-house MUC5AC ELISA [E2, E3] adapted from Takeyama and colleagues [E9]. Since no MUC5AC standard was commercially available, this ELISA produced a semi-quantitative analysis by comparing EGF-ve and AG1478 stimulated cultures to EGF+ve cultures.

**RNA extraction, cDNA synthesis and qPCR for MUC5AC**

Differentiated ALI cultures harvested on Day 28 for RNA extraction were detached by trypsinisation from the membrane, counted and stored in RNAlater (Applied Biosystems, Warrington, UK). Total RNA was extracted from stabilized cells using an RNeasy Mini kit (Qiagen, Crawley, UK) according to the manufacturer’s instructions and quantified on a Nanodrop spectrophotometer (Thermo Scientific, UK). cDNA synthesis was performed using GoScript Reverse Transcription System (Medical Supply Co., Republic of Ireland) according to the manufacturer’s protocol. For the real time PCR reaction, the DNA amplification was carried out using the Fast Start Universal SYBR Green Master (Rox) (Roche, UK) according to the manufacturers’ protocol. For each PCR reaction separate PCR Master Mixes were prepared containing either GAPDH (housekeeping gene) or MUC5AC primers (Invitrogen, Paisley, UK). Primers were designed according to the following descriptions:

**GAPDH primers -** Forward 5’ GGA GTC AAC GGA TTT GGT CG 3’ and Reverse 5’ AGG AGG CAT TGC TGA TGA TC 3’

**MUC5AC primers -** Forward 5’ TCC TTT CGT GTT GTC ACC GA 3’ localization on cDNA: 2874 bp and Reverse 5’ TCT TGA TGG CCT TGG AGC 3’ localization on cDNA: 2943 bp

A final total volume of 10μl was constituted with the addition of 1μl of cDNA into each Master Mix. Samples were placed into individual wells in duplicate in an Agilent Mx3005P 96-well detection plate system.

**Statistical Analysis**

Descriptive data was expressed as mean (standard deviation, SD) or median (interquartile range, IQR). We used one and two-way repeated measures ANOVA while, when appropriate, simultaneously adjusted for differing total cell counts (covariate) in each culture.

If in the overall model p≤ 0.05 we performed *a priori* planned comparisons using unadjusted paired t-tests. Analysis was carried out using JMP version 11 (SAS, Cary, North Carolina, USA) and GraphPad PRISM 5 (CA, USA). A p-value of <0.05 was taken as statistically significant.

**References**

E1 - Stevenson EC, Turner G, Heaney LG, et al. (1997) Bronchoalveolar lavage findings suggest two different forms of childhood asthma. Clin Exp Allergy 27(9): 1027-1035.

E2 - Parker J, Sarlang S, Thavagnanam S, et al. (2010) A 3-D well-differentiated model of paediatric bronchial epithelium demonstrates unstimulated morphological differences between asthmatic and non-asthmatic cells. Ped Res 67**:** 17**-**22.

E3 - Thavagnanam S, Parker JC, Skibinski G, et al. (2011) Effects of Interleukin-13 on mucociliary differentiation of paediatric asthmatic bronchial epithelial cells. Ped Res 69(2): 95-100.

E4 - Doherty GM, Christie SN, Skibinski G, et al. (2003) Non-bronchoscopic sampling and culture of bronchial epithelial cells in children. Clin Exp Allergy 33**:** 1221-1225.

E5 - Coyle PV, Ong GM, O'Neill HJ, et al. (2004) A touchdown nucleic acid amplification protocol as an alternative to culture backup for immunofluorescence in the routine diagnosis of acute viral respiratory tract infections. BMC Microbiol 4: 41.

E6 - Gray, TE, Guzman K, Davis CW, et al. (1996) Mucociliary differentiation of serially passaged non-asthmatic human tracheobronchial epithelial cells. Am J Respir Cell Mol Biol 14**:** 104-112.

E7 - Fulcher ML, Gabriel S, Burns KA, et al. (2005) Well-differentiated human airway epithelial cell cultures. Methods Mol Med 107**:** 183-206.

E8 - Denker BM, Nigam SK (1998) Molecular structure and assembly of the tight junction. Am J Physiol 274: F1-9.

E9 - Takeyama K, Dabbagh K, Lee HM, et al. (1999) Epidermal growth factor system regulates mucin production in airways. Proc Natl Acad Sci USA 96**:** 3081-3086.
